# Nevada

**Published:** 1897-05

**Authors:** 


*Nevada.* This State will have to raise up barriers of protection at an early date or she will find her people burdened with an additional load beyond the ability of the agriculturists of any State to sustain. We refer to the recent sale into that State, by a well-known breeder in the East, of two carloads of thoroughbred cattle from a herd known to have tuberculosis among them, and the progeny of which the owner found it difficult to sell at home without subjecting them to the tuberculin-test and the production of a certificate showing the animals to be free from tuberculosis. The date is rapidly approaching when interstate inspection must be established and a greater security furnished our stockraisers and breeders from these controllable sources of danger.

